# A randomized, observer-blinded, equivalence trial comparing two variations of Euvichol®, a bivalent killed whole-cell oral cholera vaccine, in healthy adults and children in the Philippines^☆^

**DOI:** 10.1016/j.vaccine.2018.05.102

**Published:** 2018-07-05

**Authors:** Paola Russo, Antonio D. Ligsay, Remigio Olveda, Seuk Keun Choi, Deok Ryun Kim, Ju Yeon Park, Ju Yeong Park, Khalid Ali Syed, Ayan Dey, Yang Hee Kim, Sung Hee Lee, Jayoung Kim, Yun Chon, Laura Digilio, Chan Wha Kim, Jean-Louis Excler

**Affiliations:** a Clinical Development & Regulatory, Development & Delivery Unit, International Vaccine Institute, Seoul, Republic of Korea; b National Children’s Hospital, Manila, Philippines; c Research Institute for Tropical Medicine, Manila, Philippines; d EuBiologics Co., Ltd., Seoul, Republic of Korea; e Department of Biotechnology, Korea University, Seoul, Republic of Korea; f Biostatistics & Data Management, Development & Delivery Unit, International Vaccine Institute, Seoul, Republic of Korea; g Translational Immunology Laboratory, Science Unit, International Vaccine Institute, Seoul, Republic of Korea; h MSD Wellcome Trust Hilleman Laboratories, New Delhi, India; i Program Management, Development & Delivery Unit, International Vaccine Institute, Seoul, Republic of Korea; j Development & Delivery Unit, International Vaccine Institute, Seoul, Republic of Korea

**Keywords:** ADR, Adverse Drug Reaction, AE, Adverse Event, CI, Confidence Interval, CV, Coefficient of Variation, GAVI, Gavi, The Vaccine Alliance, GMR, Geometric Mean Ratio, GMT, Geometric Mean Titer, GTFCC, Global Task Force for Cholera Control, IRB, Institutional Review Board, IVI, International Vaccine Institute, KMFDS, Korea Ministry of Food and Drug Safety, LEU, Lipopolysaccharide ELISA Unit, mITT, modified Intention-To-Treat, NCH, National Children’s Hospital, OCV, Oral Cholera Vaccine, PP, Per Protocol, PT, Preferred Term, RITM, Research Institute for Tropical Medicine, SAE, Serious Adverse Events, SOC, System Organ Class, UPT, Urine Pregnancy Test, WHA, World Health Assembly, WHO, World Health Organization, Cholera, Oral cholera vaccine, Euvichol, Equivalence, The Philippines

## Abstract

**Background:**

To contribute to the global demand for oral cholera vaccine (OCV), the production of Euvichol® was scaled up with elimination of thimerosal. To demonstrate the equivalence of the variations, a study was carried out in the Philippines.

**Methods:**

Healthy male and female adults and children in Manila were randomized to receive two doses of Euvichol® two weeks apart from either the 100L (Comparator) or the 600L (Test) variation. Primary and secondary immunogenicity endpoints were respectively geometric mean titer (GMT) of vibriocidal antibodies (two weeks post second dose) and seroconversion rate (two weeks after each dose) against O1 Inaba, Ogawa, and O139 serogroups. The GMT of vibriocidal antibodies against O1 Inaba, Ogawa, and O139 two weeks post first dose was also measured. To show the equivalence of two variations of Euvichol®, the ratio of GMT and the difference of seroconversion rate between Test and Comparator vaccines were tested with equivalence margin of [0.5, 2.0] for GMT ratio and of 15% for seroconversion rate, respectively. Safety assessment included solicited reactogenicity within 6 days after each dose and unsolicited and serious adverse events.

**Results:**

A total of 442 participants were enrolled. For the overall population, equivalence between Test and Comparator was demonstrated for vibriocidal antibody response against O1 Inaba and Ogawa serotypes and O139 serogroup in both modified intention-to-treat (mITT) and per protocol analysis, since the 95% confidence intervals (CI) of GMT to any serotypes were within the lower and upper boundary [0.5, 2.0]. Seroconversion rates after two doses also showed equivalence for O1 Inaba, Ogawa, and O139. The vaccine was safe and well tolerated, similarly between the two groups.

**Conclusion:**

The study results support the equivalence of the 600L Euvichol® to the 100L formulation in healthy children and adults. The 600L Euvichol® is safe and immunogenic in adults and children.

ClinicalTrials.gov registration number: NCT02502331.

## Highlights

• Bridging study demonstrating the equivalence of two variations of Euvichol®.
• The 600L thimerosal-free Euvichol® is safe and immunogenic in adults and children.
• The scale-up of Euvichol® allows expanding global access to oral cholera vaccine.

## 1 Introduction

With estimated 1.3–4.0 million cholera cases and 21,000–143,000 annual deaths in endemic countries [1], the World Health Organization (WHO) recommends oral cholera vaccine (OCV) use for control of both endemic and epidemic cholera [2], [3]. Following the 2011 World Health Assembly [4], an OCV stockpile has been established in 2013 for emergency responses [5]. To be utilized through the stockpile, vaccines must be WHO-prequalified (WHO PQ) [4]. Three WHO PQ OCVs are currently available: Dukoral®, Shanchol™, and Euvichol®. Shanchol™ and Euvichol® were developed following the same technology transfer from the International Vaccine Institute (IVI) initially to Shantha Biotechnics Ltd. (India) and subsequently to EuBiologics Co., Ltd. (Republic of Korea). The IVI’s formulated OCV is a whole-cell killed liquid formulation containing O1 (Inaba and Ogawa) and O139 serogroups of *Vibrio cholerae*, inactivated by heat or formalin. In Shanchol™, thimerosal was added as a preservative. Euvichol® was originally developed in 100L formulation in order to be equivalent to Shanchol™ in terms of quality, safety and immunogenicity, thus containing the same active ingredients as well as thimerosal as preservative. Both Shanchol™ and Euvichol® are presented as single-dose vials, with two doses being administered with a 2-week interval to all persons 1 year of age and older [6], [7]. Euvichol® obtained WHO PQ in December 2015 following a Phase III trial which showed non-inferiority to Shanchol™ [8], [9]. To meet the increasing OCV global demand [1], [10], as recently illustrated by the massive outbreak in Yemen [11], the manufacturing process of Euvichol® was scaled-up to 600L fermenter. Thimerosal was no longer added since not required for a single-dose vaccine [12]. The Korea Ministry of Food and Drug Safety (KMFDS) as well as WHO evaluated the process changes as minor, and thimerosal-free Euvichol® (600L) variation received WHO PQ in September 2016.

The objectives of this study were to assess safety and immunogenicity and to demonstrate the equivalence of the already WHO PQ formulation (100L fermenter, with thimerosal) to the scaled-up formulation (600L fermenter, thimerosal-free).

This study was conducted in the Philippines, where the incidence rate of cholera was estimated at 1/10,000 with 2430 annual cases [1].

## 2 Materials and methods

The clinical study (ClinicalTrials.gov NCT02502331) was approved by the Philippines Food and Drug Administration and by the Institutional Review Boards (IRB) of the National Children’s Hospital (NCH), the Research Institute for Tropical Medicine (RITM), and of IVI. The study was conducted in accordance with the ICH E8 Guideline for Good Clinical Practice and the ethical principles of the Declaration of Helsinki.

### 2.1 Study design, vaccines and participants

This was a randomized, observer-blinded, equivalence, multicenter study to assess and compare the safety and immunogenicity of the scaled-up formulation of Euvichol® (Test vaccine: 600L fermenter and thimerosal-free) with the originally licensed formulation (Comparator vaccine: 100L fermenter with thimerosal) [9].

Participants were healthy Filipino adults and children, recruited at the NCH and the RITM clinical sites in Manila. Written informed consent was obtained from eligible adult participants and from the parents or legal guardians of participants aged 1–17 years. Assent was also obtained from 7 to 17 years old children according to the 2011 Philippines National Ethics Guidelines.

Participants were stratified into adults (18–40 years old) and children (1–17 years old) cohorts and were enrolled in May-June 2016. Subjects with gastrointestinal symptoms occurring up to one week before study initiation, history of cholera or cholera vaccination as well as pregnant or lactating women were excluded. A urine pregnancy test (UPT) by urine HCG was performed at screening and, subsequently, at each of the three scheduled visits, in all women who reached the age of menarche, excluding those who had hysterectomy or bilateral ovariectomy. Women with bilateral tubal ligation underwent a UPT at each visit.

Eligible participants were randomized to one of two (Test or Comparator) vaccine groups, so that the same number of participants was randomly allocated to each of the two vaccine groups. The formulation of Euvichol® was reported previously [9]. Both vaccine variations were presented in single dose glass vials and administered orally by oral syringe in two doses (1.5 mL each) two weeks apart. Vaccines were stored at +2–8 °C. Participants were instructed not to eat one hour before and after dosing while water intake was allowed.

Screening and enrollment with randomization took place at Day 0, when enrolled participants received the first dose. The second dose was administered after two weeks (Visit 2, Day 14) with a window period of +/−3 days. Participants were followed up for two weeks after the second dose (Visit 3, Day 28 +/−3 days), observed for 30 min following vaccination, and given diary cards at Visits 1 and 2, in order to record any solicited and unsolicited adverse events (AEs) occurring up to 6 days following each vaccination. Adverse events, serious adverse events (SAEs) and concomitant medications were monitored until Day 28, end of the study. On Day 7 and Day 21 (+3 days if necessary), adult participants and parents or legal guardian of children participants were interviewed through phone call or home visit by study staff for AE monitoring. At the end of study, women of childbearing age were followed up for 3 months through monthly phone call or home visit to assess if any pregnancy had occurred.

### 2.2 Sample size, randomization and blinding

Adults (18–40 years) and children (1–17 years) participating were recruited in 1:1 ratio at the two sites. The 1–17 years age cohort was further stratified into 1–5 years (82 subjects) and 6–17 years (162 subjects) age groups to achieve balance and proportionate representation among vaccine groups (not statistically powered), so that the number of participants within each of the two children sub-cohorts is very similar in Test vs. Comparator groups. The sample size was calculated within each age strata of adults and children separately (244 children and 198 adults, respectively) to provide 90% power to show equivalence of the serotype-specific geometric mean titers (GMT) of vibriocidal antibodies between two vaccine groups using two one-sided tests with significant level of 0.025 each. The lower and upper equivalence margins for the test of the GMT ratio (GMR) were 0.5 and 2.0 when the assumed true ratio of the means is 1.00. According to the formula of sample size calculation, the coefficient of variation (CV) of untransformed vibriocidal antibody titers was 2.50 for the children cohort and 2.02 for the adult cohort, based on the previous Phase III study [9]. A 10% dropout rate was considered. The sample size was obtained using the software PASS 2008 module of Equivalence Tests for Two Means using Ratios, referring to Julious power formulae for equivalence trials with acceptance limits for unknown variance and under the assumptions of a non-central t distribution [13].

Block randomization process ensured an effective balance between interventions. Since Test and Comparator vaccines had different aluminum vial caps, the study was single-blinded to ensure evaluator’s blinding to prevent bias of assessment of adverse events.

### 2.3 Immunogenicity assessment

Blood samples for immunogenicity assessment were obtained immediately prior to first dose on Day 0, before second dose (Day 14) and 14 days post second dose (Day 28). Sera were separated and stored at −70 °C until shipment to IVI. Vibriocidal antibody assays were performed at the IVI Translational Immunology Laboratory using a microtiter technique as previously described [8], [14], [15]. The strains used in this test were *V. cholerae* O1 strain T19479 (El Tor Inaba), X25049 (El Tor Ogawa), and O139 strain CIRS 134-SR. The serum vibriocidal antibody titers were tested in duplicates and determined using the mean of two tests. The assay was repeated whenever more than two-fold difference was noted between the results of duplicates, with two operators running the test. The positive control for this study was developed using pooled high titer serum from a previous Euvichol® vaccine trial [9].

To assess the equivalence of Test and Comparator vaccines, we considered the immunogenicity in the overall population. The analysis was also performed per age cohort as secondary objective. These analyses were in accordance with the previous Euvichol® Phase III study [9], which demonstrated non-inferiority of Euvichol® to Shanchol™.

Primary immunogenicity endpoints were GMT of serum vibriocidal antibodies against Inaba and Ogawa serogroup O1 and serogroup O139 post second dose. Only the primary endpoint analysis was powered.

As secondary immunogenicity endpoint, the seroconversion rate was determined as the proportion of participants with at least 4-fold rise of O1 (Inaba and Ogawa) and O139 antibody titer two weeks post first dose (Day 14, Visit 2) and two weeks post second dose (Day 28, Visit 3) compared to baseline titer measured prior to the first vaccination. The p-value has been derived using Equivalence test with margin [−15%, +15%], considered acceptable as a general bio-equivalence margin (+/−20%) [16]. The GMT of vibriocidal antibodies against O1 Inaba, Ogawa, and O139 post first vaccine dose (Day 14, Visit 2) was an additional exploratory endpoint.

Analyses were primarily conducted to test the equivalence in antibody titers between two vaccine groups using the ratio of GMT of vibriocidal antibodies post second dose. The equivalence between the Test and Comparator vaccine groups was confirmed if the 95% confidence interval (CI) of the GMR of Test to Comparator group fell within the equivalence margins [0.5, 2.0] [17] for vibriocidal antibodies against O1 Inaba, Ogawa, or O139 [18].

For the primary analysis, the missing immunogenicity data due to withdrawn participation were not imputed, since the missing rate of primary efficacy endpoints was very low (<10%). No adjustment was used for type 1 error for multiple tests of serotypes O1 Inaba, Ogawa, and O139. The tests of O1 serotypes (Inaba and Ogawa) and of O139 were considered separately.

The modified intention-to-treat (mITT) analysis set included all participants who received a dose of investigational vaccines and had post-baseline immunogenicity data available. The per-protocol (PP) analysis set, a subset of the mITT population, included eligible participants who fully complied with all study procedures. The mITT and PP analysis sets were used for immunogenicity endpoints, as primary and secondary analysis of interest, respectively. Analysis was adjusted for study sites and age strata as covariates in a general linear model (GLM) of log scale of titer value for GMT, and in a generalized linear model with binomial distribution and identity link function for seroconversion rate difference.

We also performed a post hoc analysis excluding participants with baseline titers over 80 or 160, as commonly adopted cut-off in highly endemic settings [19], [20].

### 2.4 Safety assessment

Safety was analyzed in the overall population and in age-specific adults and children groups. The analysis set included all randomized participants who received a dose of investigational vaccines. The endpoints included the proportion of participants with solicited reactogenicity events: immediate reactions within 30 min after each vaccination and nausea/vomiting, diarrhea, headache, fatigue, myalgia, fever, and anorexia/loss of appetite within 6 days after each vaccination day collected from participants’ diary card; unsolicited AEs and SAEs occurring in entire study period. The proportion of adverse drug reactions (ADRs) was also analyzed. The list of unsolicited AEs was analyzed after coding into System Organ Class (SOC) and preferred term (PT) using MedDRA. For prior and concomitant medications, classification per drug was assessed by WHO-ATC code [21].

## 3 Results

### 3.1 Population

**Fig. 1:**
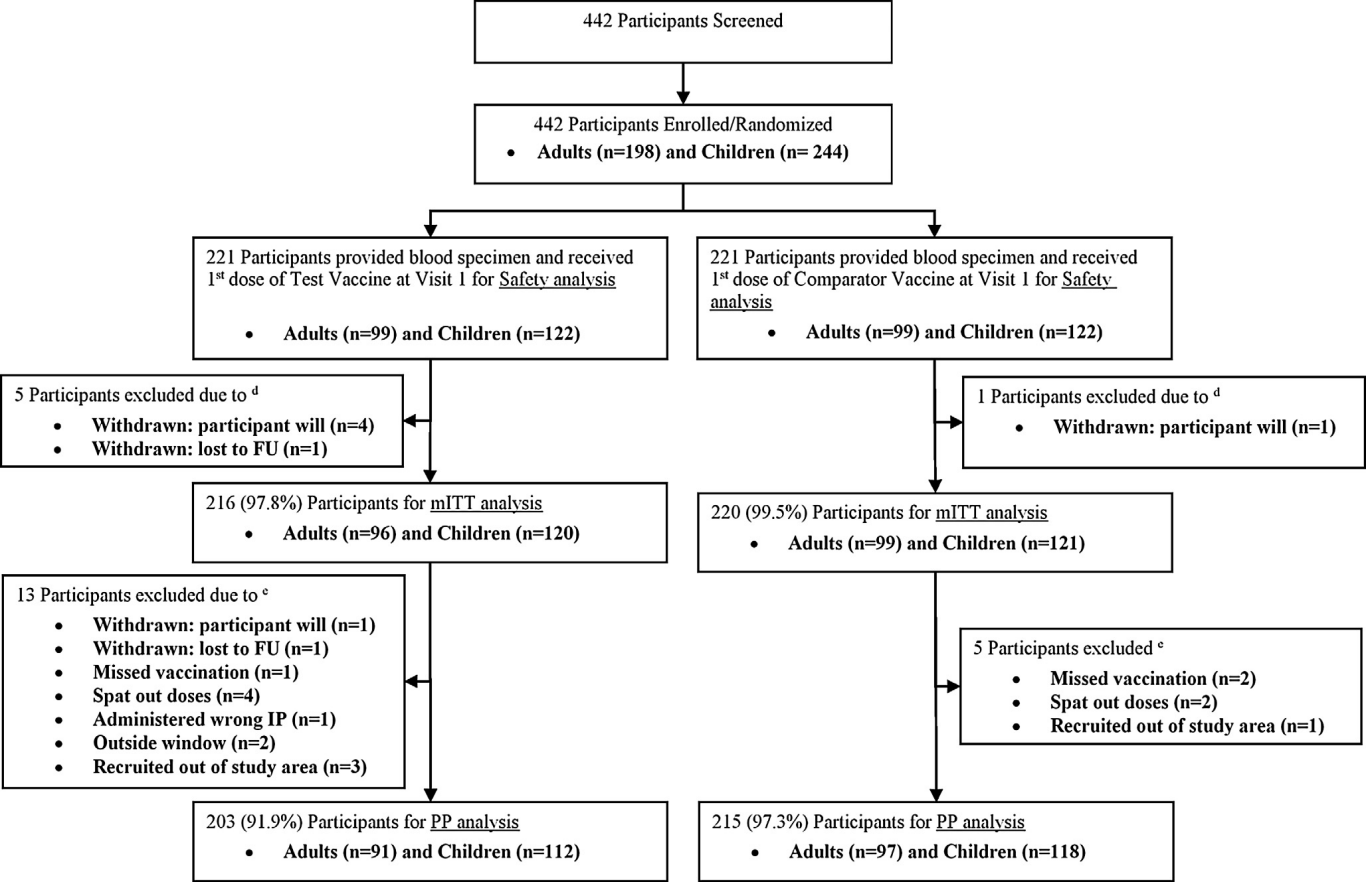
Flow Diagram of Participants Disposition (CONSORT flow diagram). *Note:* d: mITT analysis set [excluded: 3 adults (2 withdrawn by participants’ own will and 1 lost follow-up) and 2 children (withdrawn by participants’ own will) in Test vaccine group, and 1 child (withdrawn by participants’ own will) in Comparator vaccine group], e: PP analysis set [excluded: 5 adults (1 withdrawn by own will, 1 outside visit window, and 3 recruited out of study area) and 8 children (1 withdrawn due to lost to follow-up, 1 missed vaccination due to safety issue, 4 spat out the vaccine, 1 was administered wrong IP, and 1 due to outside visit window) in Test vaccine group; 2 adults (1 missed vaccination due to pregnancy and 1 was recruited out of study area) and 3 children (1 missed vaccination due to SAE and 2 spat out) in Comparator vaccine group].

A total of 442 participants were stratified into 198 adults and 244 children. The safety analysis group included 442 participants who gave consent to participate in this study, were screened, enrolled and randomized, and were administered at least one dose. Six participants from the safety analysis set with no immunogenicity assessment were excluded from the immunogenicity analysis (mITT set). Thus the number of participants in the mITT set was 436. A total of 18 participants in the mITT set were excluded from the PP set, which counted 418 participants (Fig. 1). The overall dropout rate was 5.4%. The baseline characteristics of participants did not differ significantly between vaccine groups as shown in Table 1.

**Table 1:** Baseline characteristics of the study population.

Demographic Characteristics		Test Group	Comparator Group	Total	p-value
Adults cohort		N = 99	N = 99	N = 198	
Gender	Male (%)	47 (47.5)	42 (42.4)	89 (45)	0.475
	Female (%)	52 (52.5)	57 (57.6)	109 (55)	
Age (years)	Mean (SD)	28.3 (5.9)	28.3 (6.1)	28.3 (6)	0.976
	Median (min, max)	29 (18, 40)	29 (18, 39)	29 (18, 40)	
Children cohort		N = 122	N = 122	N = 244	
Gender	Male (%)	67 (54.9)	64 (52.5)	131 (53.7)	0.700
	Female (%)	55 (45.1)	58 (47.5)	113 (46.3)	
Age (years)	Mean (SD)	8.1 (4.7)	8.8 (4.9)	8.4 (4.8)	0.290
	Median (min, max)	8 (1, 17)	9 (1, 17)	9 (1, 17)	

Baseline vibriocidal titers		Test Group	Comparator Group	p-value

All Ages		N = 216	N = 220	
O1 Inaba	GMT (95% CI)	24.01 (16.58, 34.79)	31.78 (22.62, 44.65)	0.27
O1 Ogawa	GMT (95% CI)	27.22 (18.89, 39.22)	32.39 (22.73, 46.14)	0.50
O139	GMT (95% CI)	1.73 (1.52, 1.97)	1.85 (1.59, 2.15)	0.50
Adults cohort		N = 96	N = 99	
O1 Inaba	GMT (95% CI)	52.63 (30.82, 89.87)	57.57 (35.23, 94.07)	0.81
O1 Ogawa	GMT (95% CI)	73.36 (43.97, 122.41)	50.05 (30.39, 82.41)	0.29
O139	GMT (95% CI)	1.69 (1.40, 2.04)	1.90 (1.53, 2.36)	0.42
Children cohort		N = 96	N = 99	
O1 Inaba	GMT (95% CI)	12.82 (7.86, 20.91)	19.55 (12.37, 30.89)	0.21
O1 Ogawa	GMT (95% CI)	12.31 (7.66, 19.78)	22.69 (13.83, 37.21)	0.08
O139	GMT (95% CI)	1.76 (1.47, 2.12)	1.81 (1.47, 2.24)	0.84

Baseline vibriocidal titers		Test Group	Comparator Group	p-value

All Ages		N = 216	N = 220	
O1 Inaba > 80	n (%)	78 (17.9)	88 (20.2)	0.40
O1 Ogawa > 80	n (%)	91 (20.9)	84 (19.3)	0.40
O139 > 80	n (%)	2 (0.5)	7 (1.6)	0.18
Adults cohort		N = 96	N = 99	
O1 Inaba > 80	n (%)	44 (22.6)	52 (26.7)	0.35
O1 Ogawa > 80	n (%)	54 (27.7)	47 (24.1)	0.22
O139 > 80	n (%)	0 (0)	1 (0.5)	1.00
Children cohort		N = 120	N = 121	
O1 Inaba > 80	n (%)	34 (14.1)	36 (14.9)	0.81
O1 Ogawa > 80	n (%)	37 (15.4)	37 (15.4)	0.97
O139 > 80	n (%)	2 (0.8)	6 (2.5)	0.28
All Ages		N = 216	N = 220	
O1 Inaba > 160	n (%)	58 (13.3)	56 (12.8)	0.74
O1 Ogawa > 160	n (%)	60 (13.8)	62 (14.2)	0.93
O139 > 160	n (%)	1 (0.2)	2 (0.5)	1.00
Adults cohort		N = 96	N = 99	
O1 Inaba > 160	n (%)	32 (16.4)	31 (15.9)	0.76
O1 Ogawa > 160	n (%)	37 (19.0)	35 (17.9)	0.65
O139 > 160	n (%)	0 (0)	0 (0)	NA
Children cohort		N = 120	N = 121	
O1 Inaba > 160	n (%)	26 (10.8)	25 (10.4)	0.85
O1 Ogawa > 160	n (%)	23 (9.5)	27 (11.2)	0.55
O139 > 160	n (%)	1 (0.4)	2 (0.8)	1.00

### 3.2 Immunogenicity results

Overall, Test vaccine was immunogenic in both adults and children. The equivalence of the two Euvichol® variations was confirmed on the overall analysis of combined age cohorts with a statistical power >90%. However, due to an observed GMT Coefficient of Variation higher than expected (CV range: 1.2–6.7), the immunogenicity analysis by age cohort did not reach 90% power to demonstrate the equivalence of study agents by age strata.

#### 3.2.1 Vibriocidal antibody geometric mean titers

At baseline, all ages combined, there was no significant difference in GMTs against O1 Inaba and Ogawa serotypes and O139 serogroup between Test and Comparator groups (Table 1). The overall proportions of participants in the Test group with baseline GMT >80 or >160 were respectively 18% and 13% for O1 Inaba and 21% and 14% for O1 Ogawa, with no significant difference among vaccine groups (Table 1). Baseline GMTs to O1 Inaba and Ogawa tended to be higher in the adult cohort compared to children (Table 1).

In the mITT analysis of the overall population, two weeks post second dose, the GMTs against O1 Inaba and Ogawa as well as O139 were equivalent between the Test and Comparator groups (Table 2), with a GMR of Test to Comparator group of 0.89 (95% CI of GMR: 0.66, 1.20) for O1 Inaba, 0.99 (95% CI of GMR: 0.76, 1.29) for O1 Ogawa, and GMR of 1.25 (95% CI of GMR: 0.89, 1.75) for O139. In the adult cohort, there was equivalence between the two groups for O1 Inaba and Ogawa, but not for O139 (Table 2). However, the point estimate of GMR for O139 in the adult cohort favored the Test group (GMR of 1.28; 95% CI of GMR: 0.79, 2.06). In the children cohort, GMTs of Ogawa serotype of O1 strain and O139 strain resulted in statistical equivalence between the two groups after adjustment of GMR for baseline titers, study sites, and age strata in the model (Table 2), whereas Inaba serotype of O1 strain did not show statistical equivalence (GMR of 0.69 with 95% CI of GMR: 0.46, 1.05) between the two groups (Table 2). The point estimate of GMR for O1 Inaba favored the Comparator group.

**Table 2:** Geometric Mean Titer (GMT) and Geometric Mean Ratio (GMR) two weeks post second vaccine dose – mITT set.

	Test Group (N = 214)*		Comparator Group (N = 219)*		Test/Comparator			Adjusted‡ Test/Comparator	
All Ages	GMT	95% CI	GMT	95% CI	GMR	95% CI	p-value†	GMR	95% CI
O1 Inaba	1128.10	(862.49, 1475.52)	1376.65	(1108.91, 1709.04)	0.82	(0.58, 1.16)	0.002	0.89	(0.66, 1.20)
O1 Ogawa	1457.06	(1174.97, 1806.87)	1552.60	(1255.50, 1920.00)	0.94	(0.69, 1.27)	0.000	0.99	(0.76, 1.29)
O139	14.78	(11.23, 19.46)	12.42	(9.43, 16.35)	1.19	(0.81, 1.75)	0.004	1.25	(0.89, 1.75)

By Age cohorts									

	Test Group (N = 95)*		Comparator Group (N = 98)*		Test/Comparator			Adjusted‡ Test/Comparator	
Adults cohort	GMT	95% CI of GMT	GMT	95% CI of GMT	GMR	95% CI of GMR	p-value†	GMR	95% CI of GMR

O1 Inaba	1593.21	(1140.61, 2225.39)	1354.52	(983.88, 1864.77)	1.18	(0.74, 1.86)	0.012	1.20	(0.80, 1.79)
O1 Ogawa	1688.97	(1258.17, 2267.28)	1383.56	(1054.79, 1814.82)	1.22	(0.82, 1.82)	0.008	1.10	(0.79, 1.53)
O139	11.22	(7.40, 17.02)	9.42	(6.37, 13.91)	1.19	(0.68, 2.10)	0.037	1.28	(0.79, 2.06)

	Test Group (N = 119)*		Comparator Group (N = 121)		Test/Comparator			Adjusted‡ Test/Comparator	
Children cohort	GMT	95% CI of GMT	GMT	95% CI of GMT	GMR	95% CI of GMR	p-value†	GMR	95% CI of GMR

O1 Inaba	856.37	(574.19, 1277.24)	1394.85	(1035.73, 1878.49)	0.61	(0.37, 1.01)	0.208	0.69	(0.46, 1.05)
O1 Ogawa	1295.00	(950.28, 1764.77)	1704.52	(1240.28, 2342.53)	0.76	(0.49, 1.18)	0.032	0.87	(0.60, 1.27)
O139	18.42	(12.77, 26.56)	15.54	(10.57, 22.84)	1.19	(0.70, 2.01)	0.026	1.22	(0.76, 1.95)

* The 2 participants (1 adult and 1 child) in Test group and 1 adult in Comparator group who did not have immunogenicity endpoint two weeks post second dose were excluded from the analysis.

† The p-value has been derived using Equivalence test with margin [0.5, 2.0]. The equivalence test was conducted by performing two separate tests at 2.5% significance level: (1) for lower bound, GMR < 0.5 versus GMR ≥ 0.5, and (2) for upper bound, GMR > 2.0 versus GMR ≤ 2.0. The overall p-value which is the larger of the two p-values of those tests was presented. If p-value <0.025, the two vaccine groups are equivalent.

‡ Adjusted for baseline titers and study sites in the model and additionally age strata in children cohort.

**Fig. 2:**
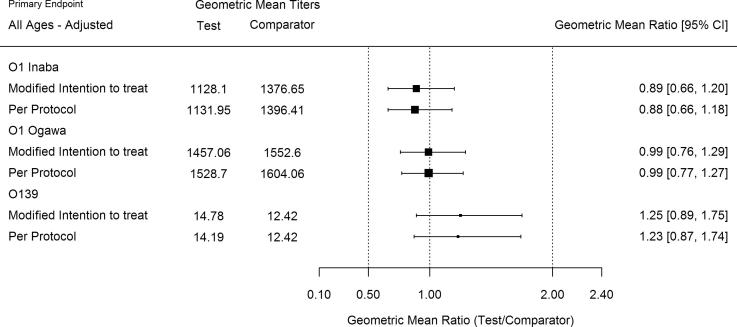
Plot of geometric mean ratio (GMR) of titers two weeks post second dose (mITT and PP analysis sets).

The analysis of the PP set two weeks post second dose in the overall population showed results consistent with the mITT analysis set (Fig. 2), with statistical equivalence between the two groups for O1 Inaba, Ogawa, and O139 strain. The PP and mITT results were also consistent when adult and children cohorts were analyzed separately (Fig. 2).

In the mITT analysis set two weeks post first dose, Test and Comparator groups were equivalent for GMT of vibriocidal antibodies against O1 Inaba (GMR of 0.85; 95% CI of GMR: 0.59, 1.22), O1 Ogawa (GMR of 0.94, with 95% CI of GMR: 0.68, 1.28), and O139 (GMR of 1.29; 95% CI of GMR: 0.89, 1.86) in the overall population (Supplementary Table 1; Supplementary Fig. 1). In the adult cohort, there was equivalence for O1 Ogawa only (GMR of 1.30, with 95% CI of GMR: 0.88, 1.91), while in the children cohort there was no equivalence for any of the serotypes of O1 and for O139 serogroup. Results in the mITT set were consistent with the PP analysis set for the overall population, with equivalence between the two vaccine groups for O1 Inaba, Ogawa and O139 (Supplementary Table 1; Supplementary Fig. 1). However, mITT and PP analysis differed by age groups. In the adult cohort, the PP analysis set showed equivalence between the two groups for O1 Inaba and Ogawa, but not for O139. In the children cohort, the PP analysis set showed equivalence between vaccine groups only for O1 Ogawa (Supplementary Table 1; Supplementary Fig. 1).

#### 3.2.2 Seroconversion rate

The analysis of seroconversion rate was primarily performed without excluding participants with GMT over 80 or 160. After the first dose in the mITT and PP set of the overall population as well as in the adult and children cohorts, equivalence of seroconversion rates for both O1 Inaba and Ogawa was demonstrated between vaccine groups. In the overall population mITT analysis set, the seroconversion rate difference for O1 Inaba was −0.33% (95% CI of difference: −7.39, 6.74) and for O1 Ogawa 2.17% (95% CI of difference: −3.79, 8.13). There was no equivalence between the two groups for O139 (difference: 8.40%; 95% CI of difference: −0.86, 17.65). However, higher proportions of seroconversion were observed in the Test group (Supplementary Table 2; Supplementary Fig. 2). Similar results were observed in the PP group (Supplementary Fig. 2).

Post second dose, the overall population mITT analysis of the seroconversion rate showed equivalence for O1 Inaba between the two groups (difference: −2.5; 95% CI of difference: −8.7, 3.8), consistent with the PP analysis set (Supplementary Table 3). In the adult cohort there was equivalence between the two groups in the mITT (difference: −2.6%; 95% CI of difference: −13.3, 8.0) but not in the PP analysis, with a higher proportion of seroconversion against O1 Inaba in the Comparator group (difference: −4.52%; 95% CI of difference: −15.3, 6.23). In the children cohort there was equivalence between the two groups both in the mITT and PP sets (Table 3 and Supplementary Table 3). For O1 Ogawa in the overall population as well as in adult and children cohorts, there was equivalence of seroconversion rates between the two groups in both the PP and mITT analyses (Table 3 and Supplementary Table 3). In particular, for the overall population in the mITT set, the seroconversion rate difference against O1 Ogawa serotype was −2.6% (95% CI of difference: −7.9, 2.7). O139 seroconversion rate showed equivalence between the two groups in the overall population by mITT (difference: 4.5%; 95% CI of difference: −4.3, 13.4) and PP analyses (Table 3 and Supplementary Table 3). In the adult cohort there was no equivalence between the two groups for O139 in the mITT set, with higher seroconversion rate in the Test group (difference: 3.9%; 95% CI of difference: −8.8, 16.5). Conversely, the PP analysis showed statistical equivalence between the two groups. In the children cohort, there was no equivalence between the two groups in both the mITT and PP analyses (Table 3 and Supplementary Table 3).

**Table 3:** Seroconversion rate difference two weeks post second vaccine dose – mITT set.

	Test Group (N = 214)‡		Comparator Group (N = 219)‡		Test – Comparator			Adjusted†	
All ages	Number of seroconverted (%)	95% CI of seroconverted	Number of seroconverted (%)	95% CI of seroconverted	Difference (%)	95% CI of Difference	p-value§	Difference (%)	95% CI of Difference
O1 Inaba	181 (84.6%)	(79.1, 88.8)	191 (87.2%)	(82.1, 91.0)	−2.6	(−9.3, 4.0)	0.000	−2.5	(−8.7, 3.8)
O1 Ogawa	192 (89.7%)	(84.9, 93.1)	200 (91.3%)	(86.9, 94.4)	−1.6	(−7.3, 4.0)	0.000	−2.6	(−7.9, 2.7)
O139	121 (56.5%)	(49.8, 63.0)	114 (52.1%)	(45.5, 58.6)	4.5	(−4.9, 13.7)	0.013	4.5	(−4.3, 13.4)

By age cohorts									

	Test Group (N = 95)‡		Comparator Group (N = 98)‡		Test – Comparator			Adjusted†	
Adults cohort	# of seroconverted (%)	95% CI of seroconverted	# of seroconverted (%)	95% CI of seroconverted	Difference (%)	95% CI of Difference	p-value§	Difference (%)	95% CI of Difference

O1 Inaba	76 (80.0%)	(70.86, 86.81)	81 (82.7%)	(74.0, 88.9)	−2.7	(−13.7, 8.4)	0.015	−2.6	(−13.3, 8.0)
O1 Ogawa	83 (87.4%)	(79.21, 92.62)	87 (88.8%)	(81.0, 93.6)	−1.4	(−10.9, 8.0)	0.003	−3.5	(−12.4, 5.3)
O139	49 (51.6%)	(41.67, 61.37)	47 (48.0%)	(38.3, 57.7)	3.6	(−10.3, 17.4)	0.055	3.9	(−8.8, 16.5)

	Test Group (N = 119)‡		Comparator Group (N = 121)		Test – Comparator			Adjusted†	

Children cohort	# of seroconverted (%)	95% CI of seroconverted	# of seroconverted (%)	95% CI of seroconverted	Difference (%)	95% CI of Difference	p-value§	Difference (%)	95% CI of Difference

O1 Inaba	105 (88.2%)	(81.2, 92.9)	110 (90.9%)	(84.5, 94.9)	−2.7	(−10.7, 5.3)	0.002	−2.4	(−9.8, 5.0)
O1 Ogawa	109 (91.6%)	(85.2, 95.4)	113 (93.4%)	(87.5, 96.619)	−1.8	(−8.9, 5.2)	0.001	−3.9	(−11.4, 3.6)
O139	72 (60.5%)	(51.5, 68.8)	67 (55.4%)	(46.5, 63.939)	5.1	(−7.3, 17.3)	0.059	5.0	(−6.8, 16.9)

‡ The 2 participants (1 adult and 1 child) in Test group and 1 adult in Comparator group who did not have immunogenicity endpoint at Visit 3 were excluded from the analysis.

§ The p-value has been derived using Equivalence test with margin [−15%, +15%]. The equivalence test was conducted by performing two separate tests at 2.5% significance level: (1) for lower bound, Difference <−15% versus Difference ≥−15%, and (2) for upper bound, Difference >+15% versus Difference ≤+15%. The overall p-value which is the higher of the two p-values of those tests was presented. If p-value <0.025, the two vaccine groups are equivalent.

† Adjusted for study sites in the model and additionally age strata in all ages combined, and in children cohort when analyzed separately.

We also analyzed the seroconversion rate difference among vaccine groups excluding participants with baseline GMT >80 or 160 (Supplementary Table 4). Results were consistent with those observed without considering the cut-off, both by age cohort and all ages combined.

### 3.3 Safety results

#### 3.3.1 Serious adverse events

No SAEs were reported in the adult cohort. Two SAEs occurred in two children within 6 days post first dose. The first SAE was severe diarrhea with fever in a 2-year old child with history of mild upper respiratory tract infection. The child completely recovered after hospitalization. The SAE was assessed as ‘possibly related’ given the temporality (24 h post-first dose of vaccine) and the absence of another identified cause. The second SAE was severe life-threatening dengue fever in a 17-year old adolescent hospitalized in the intensive care unit. The SAE was ‘unrelated’ to the study vaccine and resolved without sequelae.

#### 3.3.2 Immediate reactions, solicited adverse events and adverse drug reactions

Four children participants (3 in Test and 1 in Comparator group) experienced immediate mild reactions (nausea/vomiting) within 30 min after vaccination. All immediate reactions resolved spontaneously.

For all age cohorts, the proportion of participants (Supplementary Table 5) with solicited AEs within 6 days post first dose was 3.6% (8/221) in Test and 6.8% (15/221) in Comparator groups. Post second dose, the proportion was 2.3% (5/221) in Test and 3.2% (7/221) in Comparator groups.

In adults post first vaccination, there was a trend with more participants experiencing solicited AEs in the Comparator (12.1%, 12/99) vs. Test group (4.0%, 4/99), but this was not observed after the second vaccination, with similar frequency among groups. In children, the proportion of participants with solicited AEs after each dose was similar between groups. Overall, a trend to a reduced proportion of participants with solicited AEs after the second dose compared to the first dose was observed in all age cohorts as well as when considering separately adult and children cohorts (Supplementary Table 5). Most of solicited AEs were mild or moderate in all age cohorts as well as in adults and children cohorts, in both groups (Supplementary Table 6). Overall, in all age cohorts combined, solicited AEs were assessed as ‘definitely related’ to the study vaccines in 13 cases (54.2%) in the Test and in 28 cases (77.8%) in the Comparator groups, and ‘unlikely related’ in 3 cases (12.5%) in the Test and 4 cases (11.1%) in the Comparator groups. Two cases (5.6%) were considered ‘unrelated’ in the Comparator group (none in Test group).

All solicited AEs resolved without sequelae.

During the entire study period, 11.6% (23/198) of adults and 2.9% (7/244) of children experienced at least one adverse drug reaction (ADR). Considering all age cohorts (Supplementary Table 7), the same number of participants experienced ADR in both groups (15 subjects, 6.8%). With the exception of one child in the Test group (severe ADR), ADRs were mild or moderate.

#### 3.3.3 Unsolicited adverse events

Overall, in all age cohorts combined, the number of participants with unsolicited AEs (Supplementary Table 8) occurring within 14 days after any dose was 17 (7.7%) in the Test and 15 (6.8%) in the Comparator group. Among adults, there were 9 (9.0%) in the Test and 4 (4.0%) in the Comparator groups, whereas in the children cohort, 8 (6.6%) in the Test and 11 (9.0%) in the Comparator groups. Unsolicited AEs were mostly mild or moderate and assessed as ‘unrelated’ in the overall population and each age cohort, at any time point, irrespective of the vaccine group. Within 14 days post any dose, in all age cohorts, the category “Investigations (SOC): Body temperature increased (PT)” was the most frequent overall, occurring in 8 participants: 3 (1.4%) in Test and 5 (2.3%) in Comparator groups, followed by “Respiratory, thoracic and mediastinal disorders (SOC): Cough (PT)” in 7 participants: 5 (2.3%) in Test and 2 (0.9%) in Comparator groups.

One adult participant became pregnant during the study (negative urine pregnancy test at Visit 1, positive at Visit 2). Vaccination was discontinued (no second dose administered) and safety assessment was pursued. However, the participant was lost to follow-up.

## 4 Discussion

This study assessed the equivalence of two variations of Euvichol (scaled-up to 600L and thimerosal-free vs. 100L formulation with thimerosal). The primary immunogenicity analysis was conducted in all age cohorts combined, in accordance with the study design of the previous Euvichol® phase 3 clinical trial in The Philippines [9], which demonstrated the non-inferiority of Euvichol® vs. Shanchol™ and paved the way for the WHO pre-qualification of Euvichol®. The equivalence between the Test and Comparator groups was demonstrated as the GMR of vibriocidal antibody to any O1 serotypes and O139 serogroup were within [0.5, 2.0] of the 95% CI in the overall population after two doses compared to baseline. The analysis was also performed in adults and children separately, as, particularly in endemic settings, young children might tend to lower baseline titers [20], [22] and to a less robust immune response to OCV compared to adults [20]. The analysis by age strata demonstrated that the Test vaccine was immunogenic in both adults and children. However, the immunogenicity analysis by age cohort did not reach 90% power due to a high GMT coefficient of variation, which limits any conclusive remarks on data analyzed by age strata. Although the reasons of the GMT variability remain unclear, it might be speculated that GMTs would have shown less variability in a larger analysis set. In fact, comparing the populations in the present and previous Euvichol® studies in The Philippines, the sample size was smaller (442 subjects in the present study vs. 1263 in the pivotal study [9]) due to the selection of serum vibriocidal antibodies as primary immunogenicity endpoint, whereas the seroconversion rate was used as secondary endpoint (as opposite to the pivotal study). Conversely, the two studies shared several similarities, including safety endpoints, clinical sites, seasonality (rainy season), and schedule for vaccination and immunogenicity testing.

Another limitation to this study is the lack of unequivocal explanation for the secondary analysis results. We analyzed the immunogenicity in the overall population using the seroconversion rate after one and two doses compared to baseline. After the first dose, we observed equivalence between vaccines for Inaba and Ogawa serotypes of O1 strain but not for O139, similarly in the mITT and PP analysis sets. However, after the second dose, in the overall population, equivalence for all O1 and O139 antigens was demonstrated in both the mITT and PP analysis sets. Although there is no clear explanation for the non-equivalence of immunogenicity towards O139 between the two groups after one dose, this finding should be considered with caution, since the immunogenicity to O139 serogroup is known to be poor and highly variable compared to the immune responses elicited by O1 serogroup antigens [23], [24]. Moreover, serum vibriocidal antibodies to O1 have been regarded as an indirect immune correlate of vaccine protection, but the predictive protective value of the vibriocidal response to O139 remains unclear [23], [25]. Also, the presence of capsular polysaccharide in O139 is known to interfere with the induction or detection of vibriocidal antibody response [23].

The safety data suggest both the Test and Comparator vaccines (Euvichol® in the original 100L formulation and after scale-up to 600L fermenter and elimination [12] of thimerosal) were safe and generally well tolerated in adults and children. Moreover, safety results were similar to those observed in the previous Euvichol® study conducted in the Philippines [9].

## 5 Conclusion

The results of this study demonstrate the equivalence of thimerosal-free 600L Euvichol® with the originally licensed Euvichol® formulation (100L with thimerosal) in healthy Filipino children and adults. Based on the GMTs in the overall population, the immunogenicity of the two vaccines is equivalent for O1 Inaba and Ogawa and O139. In addition, the safety profile of the two vaccines is similar. This manufacturing of Euvichol® to 600L scale may significantly contribute to the GAVI objective of expanding the current global OCV stockpile to at least 20 million doses by 2018 and also to increase the public market supply [26].

## Conflict of interest

SKC and SHL are employees of EuBiologics Co., Ltd. Other authors declare no potential conflict of interest.
