# Supplementary material for: A randomized, observer-blinded, equivalence trial comparing two variations of Euvichol®, a bivalent killed whole-cell oral cholera vaccine, in healthy adults and children in the Philippines
Source: Vaccine. 2018 Jul 5;36(29):4317–24. doi: 10.1016/j.vaccine.2018.05.102 (PMC6026293; doi:10.1016/j.vaccine.2018.05.102)

**Supplementary Figure 2. Plot of difference of seroconversion rates at two weeks after first vaccine dose.**


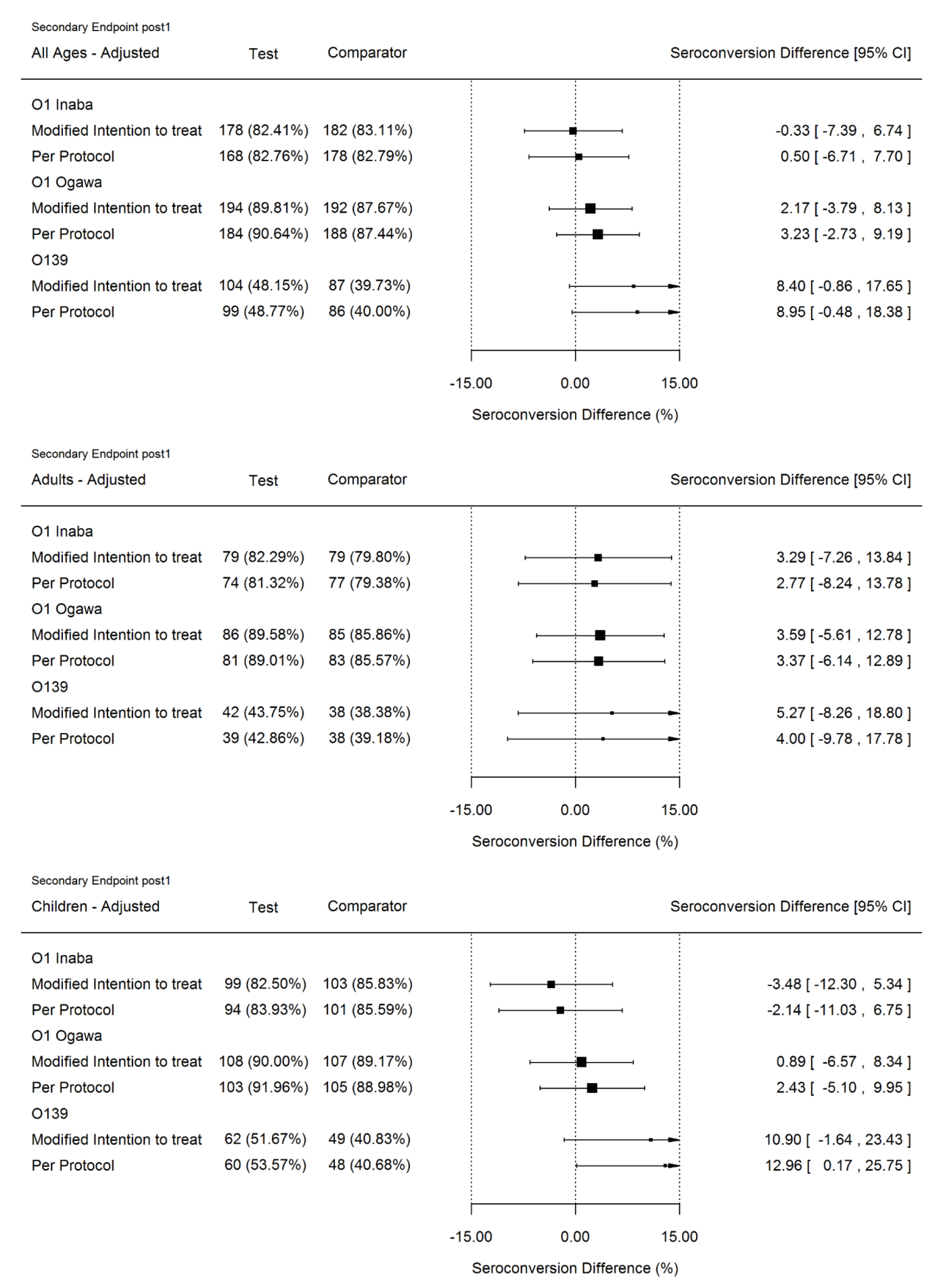

Supplement: Supplementary data 2 [file mmc2.docx]
