# Supplementary material for: A randomized, observer-blinded, equivalence trial comparing two variations of Euvichol®, a bivalent killed whole-cell oral cholera vaccine, in healthy adults and children in the Philippines
Source: Vaccine. 2018 Jul 5;36(29):4317–24. doi: 10.1016/j.vaccine.2018.05.102 (PMC6026293; doi:10.1016/j.vaccine.2018.05.102)
Supplement: Supplementary data 3 [file mmc3.docx]

**Supplementary Table 1. Exploratory immunogenicity endpoint (Geometric Mean Ratio, GMR) two weeks post first vaccine dose - mITT set**

|  | **Test Group (N=216)** | | **Comparator Group (N=219)^‡^** | | **Test/ Comparator** | | | **Adjusted ^†^ Test/ Comparator** | |
| --- | --- | --- | --- | --- | --- | --- | --- | --- | --- |
| **All Ages** | **GMT** | **95% CI of GMT** | **GMT** | **95% CI of GMT** | **GMR** | **95% CI of GMR** | **p-value*** | **GMR** | **95% CI of GMR** |
| O1 Inaba | 943.65 | (687.16, 1295.89) | 1236.20 | (931.01, 1641.44) | 0.76 | (0.50, 1.17) | 0.025 | 0.85 | (0.59, 1.22) |
| O1 Ogawa | 1378.05 | (1066.30, 1780.94) | 1557.52 | (1197.77, 2025.31) | 0.88 | (0.61, 1.28) | 0.001 | 0.94 | (0.68, 1.28) |
| O139 | 9.27 | (7.06, 12.18) | 7.50 | (5.70, 9.88) | 1.24 | 0.84, 1.82) | 0.007 | 1.29 | (0.89, 1.86) |

**^‡^**The child in Comparator group who did not have immunogenicity endpoint at Visit 2 was excluded from the analysis. *The p-value has been derived using Equivalence test with margin [0.5, 2.0]. The equivalence test was conducted by performing two separate tests at 2.5% significance level: 1) for lower bound, GMR<0.5 versus GMR≥0.5, and 2) for upper bound, GMR>2.0 versus GMR≤2.0. The overall p-value which is the higher of the two p-values of those tests was presented. If p-value <0.025, the two vaccine groups are equivalent. †Adjusted for baseline titers, study sites, and age strata in the model.

**By Age cohorts**

|  | **Test Group (N=96)** | | **Comparator Group (N=99)** | | **Test/ Comparator** | | | **Adjusted ^†^ Test/ Comparator** | |
| --- | --- | --- | --- | --- | --- | --- | --- | --- | --- |
| **Adults cohort** | **GMT** | **95% CI of GMT** | **GMT** | **95% CI of GMT** | **GMR** | **95% CI of GMR** | **p-value*** | **GMR** | **95% CI of GMR** |
| O1 Inaba | 1758.66 | (1164.56, 2655.85) | 1441.79 | (927.59, 2241.04) | 1.22 | (0.72, 2.06) | 0.032 | 1.26 | (0.78, 2.01) |
| O1 Ogawa | 2280.70 | (1637.75, 3176.06) | 1568.16 | (1066.74, 2305.26) | 1.45 | (0.94, 2.26) | 0.078 | 1.30 | (0.88, 1.91) |
| O139 | 7.58 | (4.73, 12.15) | 6.11 | (3.98, 9.39) | 1.24 | (0.71, 2.15) | 0.044 | 1.34 | (0.80, 2.23) |
|  | **Test Group (N=120)** | | **Comparator Group (N=120)^‡^** | | **Test/ Comparator** | | | **Adjusted ^†^ Test/ Comparator** | |
| **Children cohort** | **GMT** | **95% CI of GMT** | **GMT** | **95% CI of GMT** | **GMR** | **95% CI of GMR** | **p-value*** | **GMR** | **95% CI of GMR** |
| O1 Inaba | 573.48 | (331.01, 993.57) | 1088.85 | (678.61, 1747.12) | 0.53 | (0.28, 0.99) | 0.435 | 0.60 | (0.36, 1.01) |
| O1 Ogawa | 920.92 | (589.94, 1437.60) | 1548.79 | (984.14, 2437.41) | 0.59 | (0.34, 1.03) | 0.268 | 0.71 | (0.44, 1.12) |
| O139 | 10.90 | (7.15, 16.60) | 8.88 | (5.62, 14.02) | 1.23 | (0.72, 2.10) | 0.038 | 1.25 | (0.75, 2.09) |

**^‡^**The child in Comparator group who did not have immunogenicity endpoint at Visit 2 was excluded from the analysis. *The p-value has been derived using Equivalence test with margin [0.5, 2.0]. The equivalence test was conducted by performing two separate tests at 2.5% significance level: 1) for lower bound, GMR<0.5 versus GMR≥0.5, and 2) for upper bound, GMR>2.0 versus GMR≤2.0. The overall p-value which is the higher of the two p-values of those tests was presented. If p-value <0.025, the two vaccine groups are equivalent. †Adjusted for baseline titers and study site in the model and additionally age strata in children cohort.
