# Supplementary material for: A randomized, observer-blinded, equivalence trial comparing two variations of Euvichol®, a bivalent killed whole-cell oral cholera vaccine, in healthy adults and children in the Philippines
Source: Vaccine. 2018 Jul 5;36(29):4317–24. doi: 10.1016/j.vaccine.2018.05.102 (PMC6026293; doi:10.1016/j.vaccine.2018.05.102)
Supplement: Supplementary data 4 [file mmc4.docx]

**Supplementary Table 2. Secondary immunogenicity endpoint two weeks post first vaccine dose - mITT set**

|  | **Test Group (N=216)** | | **Comparator Group (N=219)^‡^** | | **Test - Comparator** | | | **Adjusted ^†^** | |
| --- | --- | --- | --- | --- | --- | --- | --- | --- | --- |
| **All ages** | **Number of seroconverted (%)** | **95% CI of seroconverted** | **Number of seroconverted (%)** | **95% CI of seroconverted** | **Difference (%)** | **95% CI of Difference** | **p-value§** | **Difference (%)** | **95% CI of Difference** |
| O1 Inaba | 178 (82.4%) | (76.78, 86.91) | 182 (83.1%) | (77.58, 87.49) | -0.7 | (-7.83, 6.43) | 0.000 | -0.33 | (-7.39, 6.74) |
| O1 Ogawa | 194 (89.8%) | (85.06, 93.18) | 192 (87.7%) | (82.66, 91.39) | 2.14 | (-3.89, 8.18) | 0.000 | 2.17 | (-3.79, 8.13) |
| O139 | 104 (48.2%) | (41.58, 54.79) | 87 (39.7%) | (33.48, 46.33) | 8.42 | (-0.90, 17.54) | 0.081 | 8.40 | (-0.86, 17.65) |

**^‡^**The 1 child in Comparator group who did not have immunogenicity endpoint at Visit 2 was excluded from the analysis. § The p-value has been derived using Equivalence test with margin [-15%, +15%]. The equivalence test was conducted by performing two separate tests at 2.5% significance level: 1) for lower bound, Difference<-15% versus Difference≥-15%, and 2) for upper bound, Difference>+15% versus Difference≤+15%. The overall p-value which is the larger of the two p-values of those tests was presented. If p-value <0.025, the two vaccine groups are equivalent. †Adjusted for study sites, and age strata in the model.

**By age cohorts**

|  | **Test Group (N=96)** | | **Comparator Group (N=99)** | | **Test – Comparator** | | | **Adjusted ^†^** | |
| --- | --- | --- | --- | --- | --- | --- | --- | --- | --- |
| **Adults cohort** | **Number of seroconverted (%)** | **95% CI of seroconverted** | **Number of seroconverted (%)** | **95% CI of seroconverted** | **Difference (%)** | **95% CI of Difference** | **p-value§** | **Difference (%)** | **95% CI of Difference** |
| O1 Inaba | 79 (82.3%) | (73.46, 88.64) | 79 (79.8%) | (70.85, 86.52) | 2.49 | (-8.61, 13.47) | 0.014 | 3.29 | (-7.26, 13.84) |
| O1 Ogawa | 86 (89.6%) | (81.88, 94.24) | 85 (85.9%) | (77.65, 91.39) | 3.72 | (-5.76, 13.16) | 0.011 | 3.59 | (-5.61, 12.78) |
| O139 | 42 (43.8%) | (34.26, 53.72) | 38 (38.4%) | (29.41, 48.23) | 5.37 | (-8.31, 18.78) | 0.084 | 5.27 | (-8.26, 18.80) |
|  | **Test Group (N=120)** | | **Comparator Group (N=120)^‡^** | | **Test – Comparator** | | | **Adjusted ^†^** | |
| **Children cohort** | **Number of seroconverted (%)** | **95% CI of seroconverted** | **Number of seroconverted (%)** | **95% CI of seroconverted** | **Difference (%)** | **95% CI of Difference** | **p-value§** | **Difference (%)** | **95% CI of Difference** |
| O1 Inaba | 99 (82.5%) | (74.72, 88.26) | 103 (85.8%) | (78.48, 90.96) | -3.33 | (-12.6, 6.01) | 0.008 | -3.48 | (-12.3, 5.34) |
| O1 Ogawa | 108 (90.0%) | (83.33, 94.19) | 107 (89.2%) | (82.34, 93.56) | 0.83 | (-7.15, 8.84) | 0.001 | 0.89 | (-6.57, 8.34) |
| O139 | 62 (51.7%) | (42.81, 60.42) | 49 (40.8%) | (32.46, 49.78) | 10.83 | (-1.75, 22.95) | 0.256 | 10.90 | (-1.64, 23.43) |

**^‡^**The 1 child in Comparator group who did not have immunogenicity endpoint at Visit 2 was excluded from the analysis. § The p-value has been derived using Equivalence test with margin [-15%, +15%]. The equivalence test was conducted by performing two separate tests at 2.5% significance level: 1) for lower bound, Difference <-15% versus Difference ≥-15%, and 2) for upper bound, Difference>+15% versus Difference ≤+15%. The overall p-value which is the higher of the two p-values of those tests was presented. If p-value <0.025, the two vaccine groups are equivalent. †Adjusted for baseline titers and study sites in the model and additionally age strata in children cohort.
