# Supplementary material for: A randomized, observer-blinded, equivalence trial comparing two variations of Euvichol®, a bivalent killed whole-cell oral cholera vaccine, in healthy adults and children in the Philippines
Source: Vaccine. 2018 Jul 5;36(29):4317–24. doi: 10.1016/j.vaccine.2018.05.102 (PMC6026293; doi:10.1016/j.vaccine.2018.05.102)
Supplement: Supplementary data 5 [file mmc5.docx]

**Supplementary Table 3. Seroconversion rate difference two weeks post second vaccine dose - PP set**

|  | **Test Group (N=203)** | | **Comparator Group (N=215)** | | **Test – Comparator** | | | **Adjusted ^†^** | |
| --- | --- | --- | --- | --- | --- | --- | --- | --- | --- |
| **All ages** | **Number of seroconverted (%)** | **95% CI of seroconverted** | **Number of seroconverted (%)** | **95% CI of seroconverted** | **Difference (%)** | **95% CI of Difference** | **p-value§** | **Difference (%)** | **95% CI of Difference** |
| O1 Inaba | 171 (84.24%) | (78.59, 88.61) | 189 (87.91%) | (82.87, 91.61) | -3.67 | (-10.4, 3.00) | 0.001 | -3.27 | (-9.41, 2.87) |
| O1 Ogawa | 182 (89.66%) | (84.70, 93.13) | 197 (91.63%) | (87.16, 94.64) | -1.97 | (-7.77, 3.69) | 0.000 | -2.82 | (-7.97, 2.32) |
| O139 | 114 (56.16%) | (49.28, 62.81) | 112 (52.09%) | (45.44, 58.68) | 4.06 | (-5.45, 13.47) | 0.012 | 4.45 | (-4.54, 13.44) |

§ The p-value has been derived using Equivalence test with margin [-15%, +15%]. The equivalence test was conducted by performing two separate tests at 2.5% significance level: 1) for lower bound, Difference <-15% versus Difference ≥-15%, and 2) for upper bound, Difference >+15% versus Difference≤+15%. The overall p-value which is the higher of the two p-values of those tests was presented. If p-value <0.025, the two vaccine groups are equivalent. †Adjusted for study sites, and age strata in the model.

**By age cohorts**

|  | **Test Group (N=91)** | | **Comparator Group (N=97)** | | **Test – Comparator** | | | **Adjusted ^†^** | |
| --- | --- | --- | --- | --- | --- | --- | --- | --- | --- |
| **Adults cohort** | **Number of seroconverted (%)** | **95% CI of seroconverted** | **Number of seroconverted (%)** | **95% CI of seroconverted** | **Difference (%)** | **95% CI of Difference** | **p-value§** | **Difference (%)** | **95% CI of Difference** |
| O1 Inaba | 72 (79.12%) | (69.68, 86.21) | 81 (83.51%) | (74.87, 89.58) | -4.38 | (-15.6, 6.78) | 0.034 | -4.52 | (-15.3, 6.23) |
| O1 Ogawa | 79 (86.81%) | (78.35, 92.29) | 86 (88.66%) | (80.83, 93.55) | -1.85 | (-11.6, 7.71) | 0.005 | -3.86 | (-13.0, 5.29) |
| O139 | 45 (49.45%) | (39.41, 59.54) | 47 (48.45%) | (38.76, 58.27) | 1.00 | (-13.0, 14.99) | 0.026 | 1.98 | (-10.9, 14.84) |
|  | **Test Group (N=112)** | | **Comparator Group (N=118)** | | **Test – Comparator** | | | **Adjusted ^†^** | |
| **Children cohort** | **Number of seroconverted (%)** | **95% CI of seroconverted** | **Number of seroconverted (%)** | **95% CI of seroconverted** | **Difference (%)** | **95% CI of Difference** | **p-value§** | **Difference (%)** | **95% CI of Difference** |
| O1 Inaba | 99 (88.39%) | (81.15, 93.09) | 108 (91.53%) | (85.10, 95.33) | -3.13 | (-11.3, 4.83) | 0.003 | -2.83 | (-10.2, 4.54) |
| O1 Ogawa | 103 (91.96%) | (85.43, 95.71) | 111 (94.07%) | (88.26, 97.10) | -2.1 | (-9.31, 4.81) | 0.001 | -3.94 | (-11.3, 3.37) |
| O139 | 69 (61.61%) | (52.36, 70.09) | 65 (55.08%) | (46.09, 63.76) | 6.52 | (-6.16, 18.88) | 0.094 | 6.14 | (-5.96, 18.24) |

§ The p-value has been derived using Equivalence test with margin [-15%, +15%]. The equivalence test was conducted by performing two separate tests at 2.5% significance level: 1) for lower bound, Difference <-15% versus Difference ≥-15%, and 2) for upper bound, Difference >+15% versus Difference ≤+15%. The overall p-value which is the higher of the two p-values of those tests was presented. If p-value <0.025, the two vaccine groups are equivalent. †Adjusted for study sites in the model and additionally age strata in children cohort.
