# Supplementary material for: A randomized, observer-blinded, equivalence trial comparing two variations of Euvichol®, a bivalent killed whole-cell oral cholera vaccine, in healthy adults and children in the Philippines
Source: Vaccine. 2018 Jul 5;36(29):4317–24. doi: 10.1016/j.vaccine.2018.05.102 (PMC6026293; doi:10.1016/j.vaccine.2018.05.102)
Supplement: Supplementary data 6 [file mmc6.docx]

**Supplementary Table 4. Seroconversion rate difference excluding participant with baseline cut-off of 80 or 160**

| **mITT set having**  **baseline titer ≤80** | |  | **Test Group** | |  | **Comparator Group** | | **Test – Comparator** | | |
| --- | --- | --- | --- | --- | --- | --- | --- | --- | --- | --- |
|  |  | **N** | **Number of seroconverted (%)** | **95% CI of seroconverted** | **N** | **Number of seroconverted (%)** | **95% CI of seroconverted** | **Difference (%)** | **95% CI of Difference** | **p-value§** |
| **Post Dose 1** | **All ages** |  |  | |  |  | |  |  |  |
|  | O1 Inaba | 138 | 121 (87.68%) | (81.16, 92.16) | 132 | 122 (92.42%) | (86.62, 95.83) | -4.74 | (-12.1, 2.59) | 0.004 |
|  | O1 Ogawa | 125 | 119 (95.20%) | (89.92, 97.78) | 135 | 127 (94.07%) | (88.74, 96.97) | 1.13 | (-4.89, 7.05) | 0.000 |
|  | O139 | 214 | 104 (48.60%) | (41.99, 55.26) | 212 | 87 (41.04%) | (34.63, 47.76) | 7.56 | (-1.87, 16.80) | 0.059 |
|  | **Adults cohort** | |  | |  |  | |  |  |  |
|  | O1 Inaba | 52 | 48 (92.31%) | (81.83, 96.97) | 47 | 43 (91.49%) | (80.07, 96.64) | 0.82 | (-10.9, 13.15) | 0.015 |
|  | O1 Ogawa | 42 | 41 (97.62%) | (87.68, 99.58) | 52 | 49 (94.23%) | (84.36, 98.02) | 3.39 | (-7.25, 13.45) | 0.016 |
|  | O139 | 96 | 42 (43.75%) | (34.26, 53.72) | 98 | 38 (38.78%) | (29.73, 48.67) | 4.97 | (-8.74, 18.44) | 0.076 |
|  | **Children cohort** | |  | |  |  | |  |  |  |
|  | O1 Inaba | 86 | 73 (84.88%) | (75.84, 90.95) | 85 | 79 (92.94%) | (85.44, 96.72) | -8.06 | (-17.9, 1.59) | 0.082 |
|  | O1 Ogawa | 83 | 78 (93.98%) | (86.66, 97.40) | 83 | 78 (93.98%) | (86.66, 97.40) | 0 | (-8.08, 8.08) | 0.001 |
|  | O139 | 118 | 62 (52.54%) | (43.60, 61.33) | 114 | 49 (42.98%) | (34.27, 52.15) | 9.56 | (-3.25, 21.93) | 0.201 |
| **Post Dose 2** | **All ages** |  |  | |  |  | |  |  |  |
|  | O1 Inaba | 136 | 127 (93.38%) | (87.90, 96.48) | 131 | 128 (97.71%) | (93.48, 99.22) | -4.33 | (-10.0, 0.91) | 0.001 |
|  | O1 Ogawa | 123 | 120 (97.56%) | (93.07, 99.17) | 135 | 133 (98.52%) | (94.76, 99.59) | -0.96 | (-5.57, 3.13) | 0.000 |
|  | O139 | 212 | 121 (57.08%) | (50.35, 63.55) | 212 | 114 (53.77%) | (47.05, 60.36) | 3.3 | (-6.11, 12.64) | 0.007 |
|  | **Adults cohort** | |  | |  |  | |  |  |  |
|  | O1 Inaba | 51 | 49 (96.08%) | (86.78, 98.92) | 46 | 44 (95.65%) | (85.47, 98.80) | 0.43 | (-9.39, 11.00) | 0.007 |
|  | O1 Ogawa | 41 | 41 (100.0%) | (91.43, 100.0) | 51 | 51 (100.0%) | (93.00, 100.0) | 0 | (NA, NA) | NA |
|  | O139 | 95 | 49 (51.58%) | (41.67, 61.37) | 97 | 47 (48.45%) | (38.76, 58.27) | 3.13 | (-10.8, 16.90) | 0.048 |
|  | **Children cohort** | |  | |  |  | |  |  |  |
|  | O1 Inaba | 85 | 78 (91.76%) | (83.96, 95.95) | 85 | 84 (98.82%) | (93.63, 99.79) | -7.06 | (-14.9, -0.39) | 0.025 |
|  | O1 Ogawa | 82 | 79 (96.34%) | (89.79, 98.75) | 84 | 82 (97.62%) | (91.73, 99.34) | -1.28 | (-8.06, 5.09) | 0.001 |
|  | O139 | 117 | 72 (61.54%) | (52.49, 69.85) | 115 | 67 (58.26%) | (49.12, 66.86) | 3.28 | (-9.21, 15.63) | 0.033 |

§ The p-value has been derived using Equivalence test with margin [-15%, +15%]. The equivalence test was conducted by performing two separate tests at 2.5% significance level: 1) for lower bound, Difference <-15% versus Difference ≥-15%, and 2) for upper bound, Difference >+15% versus Difference ≤+15%. The overall p-value which is the higher of the two p-values of those tests was presented. If p-value <0.025, the two vaccine groups are equivalent.

| **PP set having**  **baseline titer ≤80** | |  | **Test Group** | |  | **Comparator Group** | | **Test – Comparator** | | | | |
| --- | --- | --- | --- | --- | --- | --- | --- | --- | --- | --- | --- | --- |
|  |  | **N** | **Number of seroconverted (%)** | **95% CI of seroconverted** | **N** | **Number of seroconverted (%)** | **95% CI of seroconverted** | **Difference (%)** | **95% CI of Difference** | **p-value§** | |  |
| **Post Dose 1** | **All ages** |  |  | |  |  | |  |  |  | |  |
|  | O1 Inaba | 129 | 115 (89.15%) | (82.61, 93.42) | 129 | 119 (92.25%) | (86.32, 95.74) | -3.1 | (-10.5, 4.21) | 0.002 | |  |
|  | O1 Ogawa | 115 | 112 (97.39%) | (92.61, 99.11) | 131 | 123 (93.89%) | (88.41, 96.87) | 3.5 | (-2.14, 9.24) | 0.000 | |  |
|  | O139 | 201 | 99 (49.25%) | (42.42, 56.11) | 208 | 86 (41.35%) | (34.87, 48.14) | 7.91 | (-1.73, 17.34) | 0.073 | |  |
|  | **Adults cohort** | |  | |  |  | |  |  |  | |  |
|  | O1 Inaba | 49 | 45 (91.84%) | (80.81, 96.78) | 46 | 42 (91.30%) | (79.68, 96.57) | 0.53 | (-11.7, 13.17) | | 0.015 |  |
|  | O1 Ogawa | 40 | 39 (97.50%) | (87.12, 99.56) | 50 | 47 (94.00%) | (83.78, 97.94) | 3.5 | (-7.60, 13.92) | | 0.019 |  |
|  | O139 | 91 | 39 (42.86%) | (33.18, 53.11) | 96 | 38 (39.58%) | (30.38, 49.58) | 3.27 | (-10.6, 17.05) | | 0.050 |  |
|  | **Children cohort** | |  | |  |  | |  |  | |  |  |
|  | O1 Inaba | 80 | 70 (87.50%) | (78.50, 93.07) | 83 | 77 (92.77%) | (85.11, 96.64) | -5.27 | (-15.1, 4.20) | | 0.028 |  |
|  | O1 Ogawa | 75 | 73 (97.33%) | (90.79, 99.27) | 81 | 76 (93.83%) | (86.35, 97.33) | 3.51 | (-3.92, 11.23) | | 0.004 |  |
|  | O139 | 110 | 60 (54.55%) | (45.24, 63.54) | 112 | 48 (42.86%) | (34.08, 52.11) | 11.69 | (-1.43, 24.26) | | 0.309 |  |
| **Post Dose 2** | **All ages** |  |  | |  |  | |  |  |  | |  |
|  | O1 Inaba | 129 | 121 (93.80%) | (88.24, 96.82) | 129 | 127 (98.45%) | (94.52, 99.57) | -4.65 | (-10.3, 0.31) | 0.001 | |  |
|  | O1 Ogawa | 115 | 113 (98.26%) | (93.88, 99.52) | 131 | 130 (99.24%) | (95.80, 99.87) | -0.98 | (-5.40, 2.68) | 0.000 | |  |
|  | O139 | 201 | 114 (56.72%) | (49.80, 63.38) | 208 | 112 (53.85%) | (47.06, 60.49) | 2.87 | (-6.72, 12.38) | 0.006 | |  |
|  | **Adults cohort** | |  | |  |  | |  |  |  | |  |
|  | O1 Inaba | 49 | 47 (95.92%) | (86.29, 98.87) | 46 | 44 (95.65%) | (85.47, 98.80) | 0.27 | (-9.87, 10.87) | | 0.007 |  |
|  | O1 Ogawa | 40 | 40 (100.0%) | (91.24, 100.0) | 50 | 50 (100.0%) | (92.87, 100.0) | 0 | (NA, NA) | | NA |  |
|  | O139 | 91 | 45 (49.45%) | (39.41, 59.54) | 96 | 47 (48.96%) | (39.19, 58.80) | 0.49 | (-13.6, 14.53) | | 0.022 |  |
|  | **Children cohort** | |  | |  |  | |  |  | |  |  |
|  | O1 Inaba | 80 | 74 (92.50%) | (84.59, 96.52) | 83 | 83 (100.0%) | (95.58, 100.0) | -7.5 | (-15.4, -1.52) | | 0.030 |  |
|  | O1 Ogawa | 75 | 73 (97.33%) | (90.79, 99.27) | 81 | 80 (98.77%) | (93.33, 99.78) | -1.43 | (-8.06, 4.33) | | 0.001 |  |
|  | O139 | 110 | 69 (62.73%) | (53.41, 71.19) | 12 | 65 (58.04%) | (48.78, 66.76) | 4.69 | (-8.08, 17.23) | | 0.056 |  |

§ The p-value has been derived using Equivalence test with margin [-15%, +15%]. The equivalence test was conducted by performing two separate tests at 2.5% significance level: 1) for lower bound, Difference <-15% versus Difference ≥-15%, and 2) for upper bound, Difference >+15% versus Difference ≤+15%. The overall p-value which is the higher of the two p-values of those tests was presented. If p-value <0.025, the two vaccine groups are equivalent.

| **mITT set having**  **baseline titer ≤160** | |  | **Test Group** | |  | **Comparator Group** | | **Test – Comparator** | | | |
| --- | --- | --- | --- | --- | --- | --- | --- | --- | --- | --- | --- |
|  |  | **N** | **Number of seroconverted (%)** | **95% CI of seroconverted** | **N** | **Number of seroconverted (%)** | **95% CI of seroconverted** | **Difference (%)** | **95% CI of Difference** | **p-value§** | |
| **Post Dose 1** | **All ages** |  |  | |  |  | |  |  |  | |
|  | O1 Inaba | 138 | 121 (87.68%) | (81.16, 92.16) | 132 | 122 (92.42%) | (86.62, 95.83) | -4.74 | (-12.1, 2.59) | 0.004 | |
|  | O1 Ogawa | 125 | 119 (95.20%) | (89.92, 97.78) | 135 | 127 (94.07%) | (88.74, 96.97) | 1.13 | (-4.89, 7.05) | 0.000 | |
|  | O139 | 214 | 104 (48.60%) | (41.99, 55.26) | 212 | 87 (41.04%) | (34.63, 47.76) | 7.56 | (-1.87, 16.80) | 0.059 | |
|  | **Adults cohort** | |  | |  |  | |  |  |  | |
|  | O1 Inaba | 52 | 48 (92.31%) | (81.83, 96.97) | 47 | 43 (91.49%) | (80.07, 96.64) | 0.82 | (-10.9, 13.15) | 0.015 |  |
|  | O1 Ogawa | 42 | 41 (97.62%) | (87.68, 99.58) | 52 | 49 (94.23%) | (84.36, 98.02) | 3.39 | (-7.25, 13.45) | 0.016 |  |
|  | O139 | 96 | 42 (43.75%) | (34.26, 53.72) | 98 | 38 (38.78%) | (29.73, 48.67) | 4.97 | (-8.74, 18.44) | 0.076 |  |
|  | **Children cohort** | |  | |  |  | |  |  |  | |
|  | O1 Inaba | 86 | 73 (84.88%) | (75.84, 90.95) | 85 | 79 (92.94%) | (85.44, 96.72) | -8.06 | (-17.9, 1.59) | 0.082 |  |
|  | O1 Ogawa | 83 | 78 (93.98%) | (86.66, 97.40) | 83 | 78 (93.98%) | (86.66, 97.40) | 0 | (-8.08, 8.08) | 0.001 |  |
|  | O139 | 118 | 62 (52.54%) | (43.60, 61.33) | 114 | 49 (42.98%) | (34.27, 52.15) | 9.56 | (-3.25, 21.93) | 0.201 |  |
| **Post Dose 2** | **All ages** |  |  | |  |  | |  |  |  | |
|  | O1 Inaba | 136 | 127 (93.38%) | (87.90, 96.48) | 131 | 128 (97.71%) | (93.48, 99.22) | -4.33 | (-10.0, 0.91) | 0.001 | |
|  | O1 Ogawa | 123 | 120 (97.56%) | (93.07, 99.17) | 135 | 133 (98.52%) | (94.76, 99.59) | -0.96 | (-5.57, 3.13) | 0.000 | |
|  | O139 | 212 | 121 (57.08%) | (50.35, 63.55) | 212 | 114 (53.77%) | (47.05, 60.36) | 3.3 | (-6.11, 12.64) | 0.007 | |
|  | **Adults cohort** | |  | |  |  | |  |  |  | |
|  | O1 Inaba | 51 | 49 (96.08%) | (86.78, 98.92) | 46 | 44 (95.65%) | (85.47, 98.80) | 0.43 | (-9.39, 11.00) | 0.007 |  |
|  | O1 Ogawa | 41 | 41 (100.0%) | (91.43, 100.0) | 51 | 51 (100.0%) | (93.00, 100.0) | 0 | (NA, NA) | NA |  |
|  | O139 | 95 | 49 (51.58%) | (41.67, 61.37) | 97 | 47 (48.45%) | (38.76, 58.27) | 3.13 | (-10.8, 16.90) | 0.048 |  |
|  | **Children cohort** | |  | |  |  | |  |  |  | |
|  | O1 Inaba | 85 | 78 (91.76%) | (83.96, 95.95) | 85 | 84 (98.82%) | (93.63, 99.79) | -7.06 | (-14.9, -0.39) | 0.025 |  |
|  | O1 Ogawa | 82 | 79 (96.34%) | (89.79, 98.75) | 84 | 82 (97.62%) | (91.73, 99.34) | -1.28 | (-8.06, 5.09) | 0.001 |  |
|  | O139 | 117 | 72 (61.54%) | (52.49, 69.85) | 115 | 67 (58.26%) | (49.12, 66.86) | 3.28 | (-9.21, 15.63) | 0.033 |  |

§ The p-value has been derived using Equivalence test with margin [-15%, +15%]. The equivalence test was conducted by performing two separate tests at 2.5% significance level: 1) for lower bound, Difference <-15% versus Difference ≥-15%, and 2) for upper bound, Difference >+15% versus Difference ≤+15%. The overall p-value which is the higher of the two p-values of those tests was presented. If p-value <0.025, the two vaccine groups are equivalent.

| **PP set having**  **baseline titer ≤160** | |  | | **Test Group** | |  | **Comparator Group** | | **Test – Comparator** | | |
| --- | --- | --- | --- | --- | --- | --- | --- | --- | --- | --- | --- |
|  |  | **N** | | **Number of seroconverted (%)** | **95% CI of seroconverted** | **N** | **Number of seroconverted (%)** | **95% CI of seroconverted** | **Difference (%)** | **95% CI of Difference** | **p-value§** |
| **Post Dose 1** | **All ages** |  | |  | |  |  | |  |  |  |
|  | O1 Inaba | 129 | | 115 (89.15%) | (82.61, 93.42) | 129 | 119 (92.25%) | (86.32, 95.74) | -3.1 | (-10.5, 4.21) | 0.002 |
|  | O1 Ogawa | 115 | | 112 (97.39%) | (92.61, 99.11) | 131 | 123 (93.89%) | (88.41, 96.87) | 3.5 | (-2.14, 9.24) | 0.000 |
|  | O139 | 201 | | 99 (49.25%) | (42.42, 56.11) | 208 | 86 (41.35%) | (34.87, 48.14) | 7.91 | (-1.73, 17.34) | 0.073 |
|  | **Adults cohort** | | |  | |  |  | |  |  |  |
|  | O1 Inaba | | 49 | 45 (91.84%) | (80.81, 96.78) | 46 | 42 (91.30%) | (79.68, 96.57) | 0.53 | (-11.7, 13.17) | 0.015 |
|  | O1 Ogawa | | 40 | 39 (97.50%) | (87.12, 99.56) | 50 | 47 (94.00%) | (83.78, 97.94) | 3.5 | (-7.60, 13.92) | 0.019 |
|  | O139 | | 91 | 39 (42.86%) | (33.18, 53.11) | 96 | 38 (39.58%) | (30.38, 49.58) | 3.27 | (-10.6, 17.05) | 0.050 |
|  | **Children cohort** | | |  | |  |  | |  |  |  |
|  | O1 Inaba | | 80 | 70 (87.50%) | (78.50, 93.07) | 83 | 77 (92.77%) | (85.11, 96.64) | -5.27 | (-15.1, 4.20) | 0.028 |
|  | O1 Ogawa | | 75 | 73 (97.33%) | (90.79, 99.27) | 81 | 76 (93.83%) | (86.35, 97.33) | 3.51 | (-3.92, 11.23) | 0.004 |
|  | O139 | | 110 | 60 (54.55%) | (45.24, 63.54) | 112 | 48 (42.86%) | (34.08, 52.11) | 11.69 | (-1.43, 24.26) | 0.309 |
| **Post Dose 2** | **All ages** |  | |  | |  |  | |  |  |  |
|  | O1 Inaba | 129 | | 121 (93.80%) | (88.24, 96.82) | 129 | 127 (98.45%) | (94.52, 99.57) | -4.65 | (-10.3, 0.31) | 0.001 |
|  | O1 Ogawa | 115 | | 113 (98.26%) | (93.88, 99.52) | 131 | 130 (99.24%) | (95.80, 99.87) | -0.98 | (-5.40, 2.68) | 0.000 |
|  | O139 | 201 | | 114 (56.72%) | (49.80, 63.38) | 208 | 112 (53.85%) | (47.06, 60.49) | 2.87 | (-6.72, 12.38) | 0.006 |
|  | **Adults cohort** | | |  | |  |  | |  |  |  |
|  | O1 Inaba | | 49 | 47 (95.92%) | (86.29, 98.87) | 46 | 44 (95.65%) | (85.47, 98.80) | 0.27 | (-9.87, 10.87) | 0.007 |
|  | O1 Ogawa | | 40 | 40 (100.0%) | (91.24, 100.0) | 50 | 50 (100.0%) | (92.87, 100.0) | 0 | (NA, NA) | NA |
|  | O139 | | 91 | 45 (49.45%) | (39.41, 59.54) | 96 | 47 (48.96%) | (39.19, 58.80) | 0.49 | (-13.6, 14.53) | 0.022 |
|  | **Children cohort** | | |  | |  |  | |  |  |  |
|  | O1 Inaba | | 80 | 74 (92.50%) | (84.59, 96.52) | 83 | 83 (100.0%) | (95.58, 100.0) | -7.5 | (-15.4, -1.52) | 0.030 |
|  | O1 Ogawa | | 75 | 73 (97.33%) | (90.79, 99.27) | 81 | 80 (98.77%) | (93.33, 99.78) | -1.43 | (-8.06, 4.33) | 0.001 |
|  | O139 | | 110 | 69 (62.73%) | (53.41, 71.19) | 12 | 65 (58.04%) | (48.78, 66.76) | 4.69 | (-8.08, 17.23) | 0.056 |

§ The p-value has been derived using Equivalence test with margin [-15%, +15%]. The equivalence test was conducted by performing two separate tests at 2.5% significance level: 1) for lower bound, Difference <-15% versus Difference ≥-15%, and 2) for upper bound, Difference >+15% versus Difference ≤+15%. The overall p-value which is the higher of the two p-values of those tests was presented. If p-value <0.025, the two vaccine groups are equivalent.
