# Supplementary material for: A randomized, observer-blinded, equivalence trial comparing two variations of Euvichol®, a bivalent killed whole-cell oral cholera vaccine, in healthy adults and children in the Philippines
Source: Vaccine. 2018 Jul 5;36(29):4317–24. doi: 10.1016/j.vaccine.2018.05.102 (PMC6026293; doi:10.1016/j.vaccine.2018.05.102)
Supplement: Supplementary data 7 [file mmc7.docx]

**Supplementary Table 5. Summary of proportion of participants with solicited adverse events**

|  | **Test Group** | | | **Comparator Group** | | |
| --- | --- | --- | --- | --- | --- | --- |
| **Within 6 days after first dose** | **Number of participants analyzed** | **Number of participants who had AEs (%)** | **95% CI** | **Number of participants analyzed** | **Number of participants who had AEs (%)** | **95% CI** |
| All age cohorts | 221 | 8 (3.6%) | (1.85, 6.98) | 221 | 15 (6.8%) | (4.16, 10.90) |
| Adults cohort | 99 | 4 (4%) | (1.58, 9.93) | 99 | 12 (12.1%) | (7.07, 20.00) |
| Children cohort | 122 | 4 (3.3%) | (1.28, 8.13) | 122 | 3 (2.5%) | (0.84, 6.98) |
| **Within 6 days after second dose** |  | **Number of participants (%)** | **95% CI** |  | **Number of participants (%)** | **95% CI** |
| All age cohorts | 215 | 5 (2.3%) | (1.00, 5.33) | 218 | 7 (3.2%) | (1.56, 6.48) |
| Adults cohort | 96 | 3 (3.1%) | (1.07, 8.79) | 98 | 4 (4.1%) | (1.60, 10.03) |
| Children cohort | 119 | 2 (1.7%) | (0.46, 5.92) | 120 | 3 (2.5%) | (0.85, 7.09) |
| **Within 6 days after any dose** |  | **Number of participants (%)** | **95% CI** |  | **Number of participants (%)** | **95% CI** |
| All age cohorts | 221 | 13 (5.9%) | (3.47, 9.80) | 221 | 20 (9.1%) | (5.93, 13.56) |
| Adults cohort | 99 | 7 (7%) | (3.47, 13.88) | 99 | 14 (14.1%) | (8.61, 22.35) |
| Children cohort | 122 | 6 (4.9%) | (2.27, 10.32) | 122 | 6 (4.9%) | (2.27, 10.32) |
