# Supplementary material for: A randomized, observer-blinded, equivalence trial comparing two variations of Euvichol®, a bivalent killed whole-cell oral cholera vaccine, in healthy adults and children in the Philippines
Source: Vaccine. 2018 Jul 5;36(29):4317–24. doi: 10.1016/j.vaccine.2018.05.102 (PMC6026293; doi:10.1016/j.vaccine.2018.05.102)
Supplement: Supplementary data 8 [file mmc8.docx]

**Supplementary Table 6. Severity of solicited adverse events.**

|  | **Test Group** | | | **Comparator Group** | | |
| --- | --- | --- | --- | --- | --- | --- |
|  | **Number of events (%)** | | | **Number of events (%)** | | |
|  | **Adults** | **Children** | **All age cohorts** | **Adults** | **Children** | **All age cohorts** |
| **Within 6 days after first dose** | (n=99) | (n=122) | (n=221) | (n=99) | (n=122) | (n=221) |
| Grade 1 (Mild) | 6 (50.0%) | 2 (40.0%) | 8 (47.1%) | 19 (82.6%) | 2 (66.7%) | 21 (80.8%) |
| Grade 2 (Moderate) | 6 (50.0%) | 1 (20.0%) | 7 (41.2%) | 4 (17.4%) | 0 (0.0%) | 4 (15.4%) |
| Grade 3 (Severe) | 0 (0.0%) | 2 (40.0%) | 2 (11.8%) | 0 (0.0%) | 0 (0.0%) | 0 (0.0%) |
| Grade 4 (Potential Life Threatening) | 0 (0.0%) | 0 (0.0%) | 0 (0.0%) | 0 (0.0%) | 1 (33.3%) | 1 (3.9%) |
| **Total** | 12 (100%) | 5 (100%) | 17 (100%) | 23 (100%) | 3 (100%) | 26 (100%) |
| **Within 6 days after second dose** | (n=96) | (n=119) | (n=215) | (n=98) | (n=120) | (n=218) |
| Grade 1 (Mild) | 2 (40.0%) | 2 (100.0%) | 4 (57.1%) | 7 (100.0%) | 1 (33.3%) | 8 (80.0%) |
| Grade 2 (Moderate) | 2 (40.0%) | 0 (0.0%) | 2 (28.6%) | 0 (0.0%) | 1 (33.3%) | 1 (10.0%) |
| Grade 3 (Severe) | 1 (20.0%) | 0 (0.0%) | 1 (14.3%) | 0 (0.0%) | 1 (33.3%) | 1 (10.0%) |
| Grade 4 (Potential Life Threatening) | 0 (0.0%) | 0 (0.0%) | 0 (0.0%) | 0 (0.0%) | 0 (0.0%) | 0 (0.0%) |
| **Total** | 5 (100%) | 2 (100%) | 7 (100%) | 7 (100%) | 3 (100%) | 10 (100%) |
| **Within 6 days after any dose** | (n=99) | (n=122) | (n=221) | (n=99) | (n=122) | (n=221) |
| Grade 1 (Mild) | 8 (47.1%) | 4 (57.1%) | 12 (50.0%) | 26 (86.7%) | 3 (50.0%) | 29 (80.6%) |
| Grade 2 (Moderate) | 8 (47.1%) | 1 (14.3%) | 9 (37.5%) | 4 (13.3%) | 1 (16.7%) | 5 (13.9%) |
| Grade 3 (Severe) | 1 (5.9%) | 2 (28.6%) | 3 (12.5%) | 0 (0.0%) | 1 (16.7%) | 1 (2.8%) |
| Grade 4 (Potential Life Threatening) | 0 (0.0%) | 0 (0.0%) | 0 (0.0%) | 0 (0.0%) | 1 (16.7%) | 1 (2.8%) |
| **Total** | 17 (100%) | 7 (100%) | 24 (100%) | 30 (100%) | 6 (100%) | 36 (100%) |
