# Supplementary material for: A randomized, observer-blinded, equivalence trial comparing two variations of Euvichol®, a bivalent killed whole-cell oral cholera vaccine, in healthy adults and children in the Philippines
Source: Vaccine. 2018 Jul 5;36(29):4317–24. doi: 10.1016/j.vaccine.2018.05.102 (PMC6026293; doi:10.1016/j.vaccine.2018.05.102)
Supplement: Supplementary data 9 [file mmc9.docx]

**Supplementary Table 7. Summary of adverse drug reactions**

| **SUMMARY of ADRs** | | **Test Group (N=99)** | | **Comparator Group (N=99)** | |
| --- | --- | --- | --- | --- | --- |
| **ADRs during entire study period** | |  |  |  |  |
|  | | **Number of AEs** | **Number of Participants (%)** | **Number of AEs** | **Number of Participants (%)** |
| **Adult Cohort** |  | **18** | **9 (9.1%)** | **31** | **14 (14.1%)** |
| Severity: | Grade 1 (Mild) | 8 | 5 (5.1%) | 27 | 14 (14.1%) |
|  | Grade 2 (Moderate) | 10 | 7 (7.1%) | 4 | 3 (3.0%) |
|  | Grade 3 (Severe) | 0 | 0 (0.0%) | 0 | 0 (0.0%) |
|  | Grade 4 (Potential Life Threatening) | 0 | 0 (0.0%) | 0 | 0 (0.0%) |
| Relatedness: | Unrelated | 0 | 0 (0.0%) | 0 | 0 (0.0%) |
|  | Unlikely | 0 | 0 (0.0%) | 0 | 0 (0.0%) |
|  | Possibly | 7 | 5 (5.1%) | 2 | 2 (2.0%) |
|  | Probably | 0 | 0 (0.0%) | 2 | 1 (1.01%) |
|  | Definitely | 11 | 4 (4.0%) | 27 | 11 (11.1%) |

| **SUMMARY of ADRs** | | **Test Group (N=122)** | | **Comparator Group (N=122)** | |
| --- | --- | --- | --- | --- | --- |
| **ADRs during entire study period** | | **Number of AEs** | **Number of Participants (%)** | **Number of AEs** | **Number of Participants (%)** |
| **Children Cohort** |  | **7** | **6 (4.9%)** | **1** | **1 (0.8%)** |
| Severity: | Grade 1 (Mild) | 4 | 4 (3.3%) | 1 | 1 (0.8%) |
|  | Grade 2 (Moderate) | 1 | 1 (0.8%) | 0 | 0 (0.0%) |
|  | Grade 3 (Severe) | 2 | 1 (0.8%) | 0 | 0 (0.0%) |
|  | Grade 4 (Potential Life Threatening) | 0 | 0 (0.0%) | 0 | 0 (0.0%) |
| Relatedness: | Unrelated | 0 | 0 (0.0%) | 0 | 0 (0.0%) |
|  | Unlikely | 0 | 0 (0.0%) | 0 | 0 (0.0%) |
|  | Possibly | 4 | 3 (2.5%) | 0 | 0 (0.0%) |
|  | Probably | 1 | 1 (0.8%) | 0 | 0 (0.0%) |
|  | Definitely | 2 | 2 (1.6%) | 1 | 1 (0.8%) |

| **SUMMARY of ADRs** | | **Test Group (N=221)** | | **Comparator Group (N=221)** | |
| --- | --- | --- | --- | --- | --- |
| **ADRs during entire study period** | | **Number of AEs** | **Number of Participants (%)** | **Number of AEs** | **Number of Participants (%)** |
| **All age Cohorts** |  | **25** | **15 (6.8%)** | **32** | **15 (6.8%)** |
| Severity: | Grade 1 (Mild) | 12 | 9 (4.1%) | 28 | 15 (6.8%) |
|  | Grade 2 (Moderate) | 11 | 8 (3.6%) | 4 | 3 (1.4%) |
|  | Grade 3 (Severe) | 2 | 1 (0.5%) | 0 | 0 (0.0%) |
|  | Grade 4 (Potential Life Threatening) | 0 | 0 (0.0%) | 0 | 0 (0.0%) |
| Relatedness: | Unrelated | 0 | 0 (0.0%) | 0 | 0 (0.0%) |
|  | Unlikely | 0 | 0 (0.0%) | 0 | 0 (0.0%) |
|  | Possibly | 11 | 8 (3.6%) | 2 | 2 (0.9%) |
|  | Probably | 1 | 1 (0.5%) | 2 | 1 (0.5%) |
|  | Definitely | 13 | 6 (2.7%) | 28 | 12 (5.4%) |
