# Supplementary material for: A randomized, observer-blinded, equivalence trial comparing two variations of Euvichol®, a bivalent killed whole-cell oral cholera vaccine, in healthy adults and children in the Philippines
Source: Vaccine. 2018 Jul 5;36(29):4317–24. doi: 10.1016/j.vaccine.2018.05.102 (PMC6026293; doi:10.1016/j.vaccine.2018.05.102)
Supplement: Supplementary data 10 [file mmc10.docx]

**Supplementary Table 8. Summary of unsolicited AEs**

|  | **Test Group (N=221)** | | **Comparator Group (N=221)** | |
| --- | --- | --- | --- | --- |
| **Within 14 days after first dose** | **Number of participants (%)** | **95% CI** | **Number of participants (%)** | **95% CI** |
| All age cohorts | 10 (4.5%) | (2.48, 8.13) | 7 (3.2%) | (1.54, 6.39) |
| Adults cohort | 7 (7.1%) | (3.47, 13.88) | 4 (4.0%) | (1.58, 9.93) |
| Children cohort | 3 (2.5%) | (0.84, 6.98) | 3 (2.5%) | (0.84, 6.98) |
| **Within 14 days after second dose** |  |  |  |  |
| All age cohorts | 7 (3.3%) | (1.59, 6.57) | 10 (4.6%) | (2.51, 8.24) |
| Adults cohort | 2 (2.1%) | (0.57, 7.28) | 2 (2.0%) | (0.56, 7.14) |
| Children cohort | 5 (4.2%) | (1.81, 9.46) | 8 (6.67%) | (3.42, 12.61) |
| **Within 14 days after any dose** |  |  |  |  |
| All age cohorts | 17 (7.7%) | (4.86, 11.97) | 15 (6.8%) | (4.16, 10.90) |
| Adults cohort | 9 (9.1%) | (4.86, 16.38) | 4 (4.0%) | (1.58, 9.93) |
| Children cohort | 8 (6.6%) | (3.36, 12.41) | 11 (9.0%) | (5.11, 15.43) |
